# Toward nanofluids of ultra-high thermal conductivity

**DOI:** 10.1186/1556-276X-6-153

**Published:** 2011-02-18

**Authors:** Liqiu Wang, Jing Fan

**Affiliations:** 1 Department of Mechanical Engineering, The University of Hong Kong, Pokfulam Road, Hong Kong

## Abstract

The assessment of proposed origins for thermal conductivity enhancement in nanofluids signifies the importance of particle morphology and coupled transport in determining nanofluid heat conduction and thermal conductivity. The success of developing nanofluids of superior conductivity depends thus very much on our understanding and manipulation of the morphology and the coupled transport. Nanofluids with conductivity of upper Hashin-Shtrikman (H-S) bound can be obtained by manipulating particles into an interconnected configuration that disperses the base fluid and thus significantly enhancing the particle-fluid interfacial energy transport. Nanofluids with conductivity higher than the upper H-S bound could also be developed by manipulating the coupled transport among various transport processes, and thus the nature of heat conduction in nanofluids. While the direct contributions of ordered liquid layer and particle Brownian motion to the nanofluid conductivity are negligible, their indirect effects can be significant via their influence on the particle morphology and/or the coupled transport.

## Introduction

Nanofluids are a new class of fluids engineered by dispersing nanometer-size structures (particles, fibers, tubes, droplets, etc.) in base fluids. The very essence of nanofluids research and development is to enhance fluid macroscopic and system-scale properties through manipulating microscopic physics (structures, properties, and activities) [1,2]. One of such properties is the thermal conductivity that characterizes the strength of heat conduction and has become a research focus of nanofluid society in the last decade [1-9].

The importance of high-conductivity nanofluids cannot be overemphasized. The success of effectively developing such nanofluids depends very much on our understanding of mechanism responsible for the significant enhancement of thermal conductivity. Both static and dynamic reasons have been proposed for experimental finding of significant conductivity enhancement [1-9]. The former includes the nanoparticle morphology [10,11] and the liquid layering at the liquid-particle interface [12-17]. The latter contains the coupled (cross) transport [18-20] and the nanoparticle Brownian motion [21-26]. Here, the effect of particle morphology contains those from the particle shape, connectivity among particles (including and generalizing the nanoparticle clustering/aggregating in the literature [10,11]), and particle distribution in nanofluids. This short review aims for a concise assessment of these contributions, thus identifying the future research needs toward nanofluids of high thermal conductivity. The readers are referred to, for example, [1-9] for state-of-the-art expositions of major advances on the synthesis, characterization, and application of nanofluids.

### Static mechanisms

#### Morphology

The nanoparticle morphology in nanofluids can vary from a well-dispersed configuration in base fluids to a continuous phase of interconnected configuration. Such a morphology variation will change nanofluid's effective thermal conductivity significantly [27-32], a phenomenon credited to the particle clustering/aggregating in the literature [1-9]. This appears obvious because the nanofluid's effective conductivity stems mainly from the contribution of continuous phase that constitutes the continuous path for thermal flow [27,28]. Although particle clustering/aggregating offers a way of changing particle morphology, it is not necessarily an effective means. The research should thus focus not only on the clustering/aggregating, but also on the general ways of varying morphology.

Given that nanofluid thermal conductivity depends heavily on the particle morphology, its lower and upper bounds can be completely determined by the volume fractions and conductivities of the two phases. These bounds have been well developed based on the classical effective-medium theory and termed as the Hashin-Shtrikman (H-S) bounds [33],

(1) $$k_{\text{e}}/k_{\text{f}}=1+\frac{3\varphi (k_{\text{p}}/k_{\text{f}}-1)}{k_{\text{p}}/k_{\text{f}}+2-\varphi (k_{\text{p}}/k_{\text{f}}-1)},$$

(2) $$k_{\text{e}}/k_{\text{f}}=k_{\text{p}}/k_{\text{f}}\left[1-\frac{3\left(1-\varphi \right)\left(k_{\text{p}}/k_{\text{f}}-1\right)}{3k_{\text{p}}/k_{\text{f}}-\varphi \left(k_{\text{p}}/k_{\text{f}}-1\right)}\right].$$

Here *k*_p_, *k*_f_, and *k*_e _are the conductivities of particle, base fluid, and nanofluid, respectively, and *φ *is the particle volume fraction. For the case of *k*_p_/*k*_f _≥1, Equations (1) and (2) give the lower and the upper bounds for nanofluid effective thermal conductivity, corresponding to the two limiting morphologies where the liquid serves as the continuous phase for the lower bound and the particle disperses the liquid for the upper bound, respectively. When *k*_p_/*k*_f _≤1, their roles are interchanged, so that Equations (1) and (2) provide the upper and the lower bounds, respectively. Therefore, the upper bound always takes a configuration (morphology) where the continuous phase is made of the higher-conductivity material.

The morphology dependence of nanofluid's conductivity has been recently examined in detail by either of the two approaches: the constructal approach [1,2,29-32] and the scaling-up by the volume average [1,2,27,28]. Such studies not only confirm the features captured in the H-S bounds but also uncover the microscopic mechanism responsible for the morphology dependence of nanofluid's conductivity. As higher-conductivity particles interconnect each other and disperse the lower-conductivity base fluid into a dispersed phase, the interfacial energy transport between particle and base fluid becomes enhanced significantly such that the nanofluid's conductivity takes its value of upper H-S bound (Fan J and Wang LQ: Heat conduction in nanofluids: structure-property correlation, submitted).

**Figure 1:**
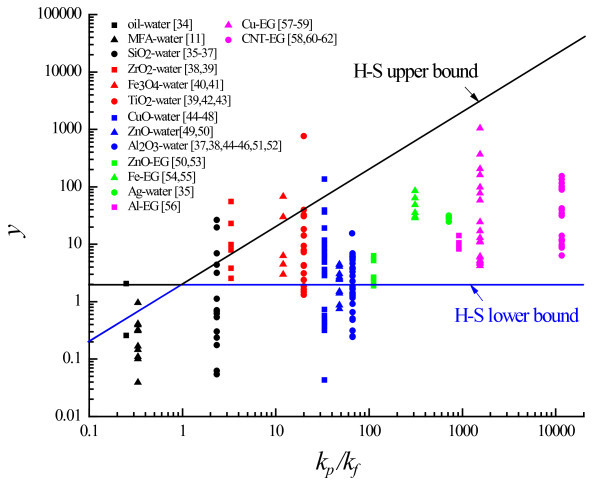
**Comparison of experimental data with H-S bounds**.

**Figure 2:**
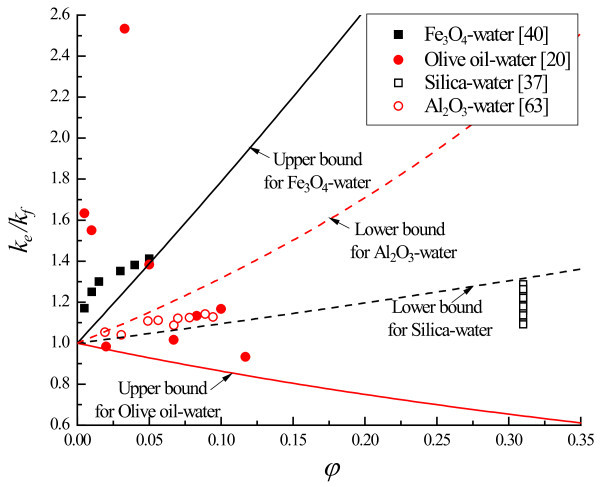
**Comparison of effective thermal conductivity between experimental data and H-S bounds**.

Figures 1 and 2 compare the experimental data of nanofluid thermal conductivity [11,20,34-63] with the H-S bounds [33]. For a concise comparison in Figure 1, the H-S bounds (Equations 1 and 2) are rewritten in the form of

(3) y=2,

and

(4) $$y=2\frac{k_{\text{p}}}{k_{\text{f}}},$$

where

(5) $$y\equiv -\frac{\left(k_{\text{p}}/k_{\text{f}}\right)\left(k_{\text{e}}/k_{\text{f}}-1\right)-\varphi \left(k_{\text{p}}/k_{\text{f}}-1\right)\left(k_{\text{e}}/k_{\text{f}}\right)}{\left(k_{\text{e}}/k_{\text{f}}-1\right)-\varphi \left(k_{\text{p}}/k_{\text{f}}-1\right)}.$$

As *k*_p_/*k*_f _moves away from the unity along both directions, the separation between the upper and lower H-S bounds becomes pronounced (Figures 1 and 2) so that the room for manipulating nanofluid conductivity via changing the particle morphology becomes more spacious. The H-S bounds are respected by some nanofluids for which their thermal conductivity is strongly dependent on particle morphology, such as whether nanoparticles stay well-dispersed in the base fluid, form aggregates, or assume a configuration of continuous phase that disperses the fluid into a dispersed phase (Figure 1). There are thermal conductivity data that fall outside the H-S bounds (Figures 1 and 2).

#### Ordered liquid layer

Both experimental and theoretical evidences have been reported of the presence of ordered liquid layer near a solid surface by which the atomic structure of the liquid layer is significantly more ordered than that of bulk liquid [64-67]. For example, two layers of icelike structures are experimentally observed to be strongly bounded to the crystal surface on a crystal-water interface, followed by two diffusive layers with less significant ordering [65]. Three ordered water layers have also been observed numerically on the Pt (111) surface [64].

The study is very limited regarding why and how these ordered liquid layers are formed. There is also a lack of detailed examination of properties of these layers, such as their thermal conductivity and thickness. Since ordered crystalline solids have normally much higher thermal conductivity than liquids, the thermal conductivity of such liquid layers is believed to be better than that of bulk liquid. The thickness *h *of such liquid layers around the solid surface can be estimated by [17]

(6) $$h=\frac{1}{\sqrt{3}}{\left(\frac{4M_{\text{f}}}{\rho_{\text{f}}N_{\text{a}}}\right)}^{1/3},$$

where *N*_a _is the Avogadro's number, and *ρ*_f _and *M*_f _are the density and the molecular weight of base fluids, respectively. The liquid layer thickness is thus 0.28 nm for water-based nanofluids, which agrees with that from experiments and molecular dynamic simulation on the order of magnitude.

**Figure 3:**
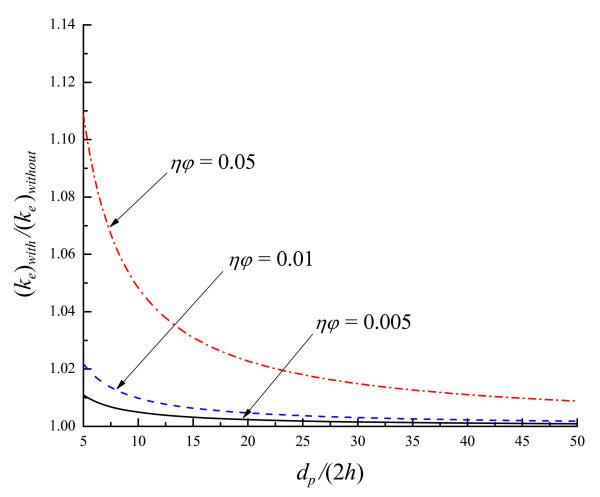
**Variations of (*k*_e_)_with_/(*k*_e_)_without _with *ηφ *and *d*_P_/(2*h*)**.

The presence of liquid layers could thus upgrade the nanofluid effective thermal conductivity via augmenting the particle effective volume fraction. For an estimation of an upper limit for this effect, assume that the thickness and the conductivity of the liquid layer are 0.5 nm and the same as that of the solid particle, respectively. For spherical particles of diameter *d*_p_, Equation (1) offers the conductivity ratio with and without this effect:

(7) $$\frac{{\left(k_{\text{e}}\right)}_{\text{with}}}{{\left(k_{\text{e}}\right)}_{\text{without}}}=\frac{1+2\eta \varphi {\left(1+2h/d_{\text{p}}\right)}^{3}}{1-\eta \varphi {\left(1+2h/d_{\text{p}}\right)}^{3}}\times \frac{1-\eta \varphi }{1+2\eta \varphi }.$$

where *η *= (*k*_p _- *k*_f_)/(*k*_p _+ 2*k*_f_). The variation of (*k*_e_)_with_/(*k*_e_)_without _with *ηφ *and *d*_p_/2*h *is illustrated in Figure 3, showing that the liquid-layering effect is important only when *ηφ *is large and *d*_p_/2*h *is small. This is normally not the case for practical nanofluids. For Cu-in-water nanofluids (η ≈ 1), for example, (*k*_e_)_with_/(*k*_e_)_without _≈ 1.005 with *φ *= 0.5% and *d*_p _= 10 nm.

**Figure 4:**
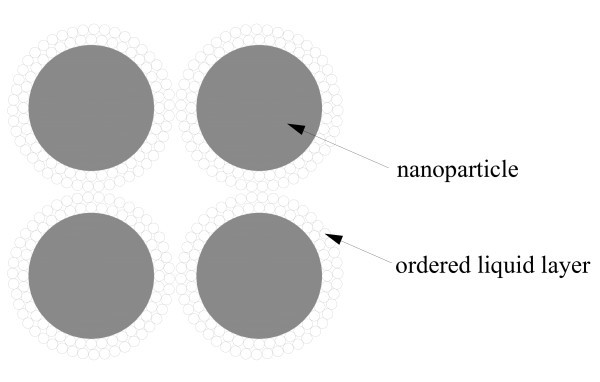
**Ordered liquid layer in promoting the formation of interconnected particle morphology**.

Although the liquid layers offer insignificant conductivity enhancement through augmenting the particle volume fraction, their presence do facilitate the formation of particle network by relaxing the requirement of particle physical contact with each other (Figure 4). This will promote the formation of interconnected particle morphology, and thus upgrade the nanofluid thermal conductivity toward its upper bound through the morphology effect.

### Dynamic mechanisms

#### Coupled transport

In a nanofluid system, normally, there are two or more transport processes that occur simultaneously. Examples are the heat conduction in dispersed phase, heat conduction in continuous phase, mass transport, and chemical reactions either among the nanoparticles or between the nanoparticles and the base fluid. These processes may couple (interfere) and cause new induced effects of flows occurring without or against its primary thermodynamic driving force, which may be a gradient of temperature, or chemical potential, or reaction affinity. Two classical examples of coupled transport are the Soret effect (also known as thermodiffusion or thermophoresis) in which directed motion of particles or macromolecules is driven by thermal gradient and the Dufour effect that is an induced heat flow caused by the concentration gradient.

While the coupled transport is well recognized to be very important in thermodynamics [68], it has not been well appreciated yet in the nanofluid society. The first attempts of examining the effect of coupled transport on nanofluid heat conduction have been recently made in some studies [1,2,9,18], which are briefly outlined here. With the coupling between the heat conduction in the fluid and particle phases denoted by *β *and *σ-*phases, respectively, the temperature *T *obeys the following energy equations [1,2]

(8) $$\gamma_{\beta }\frac{\partial T_{\beta }}{\partial t}=k_{\beta \beta }\Delta T_{\beta }+k_{\beta \sigma }\Delta T_{\sigma }+ha_{\upsilon }\left(T_{\sigma }-T_{\beta }\right)$$

and

(9) $$\gamma_{\sigma }\frac{\partial T_{\sigma }}{\partial t}=k_{\sigma \sigma }\Delta T_{\sigma }+k_{\sigma \beta }\Delta T_{\beta }-ha_{\upsilon }\left(T_{\sigma }-T_{\beta }\right)$$

where *T *is the temperature; subscripts *β *and *σ *refer to the *β *and *σ-*phases, respectively. *γ*_*β *_= (1 - *φ*)(*ρc*)*_β_*and *γ*_*σ *_= *φ*(*ρc*)_*σ *_are the effective thermal capacities of *β *and *σ-*phases, respectively, with *ρ *and *c *as the density and the specific heat. *φ *is the volume fraction of the *σ-*phase. *h *and *a*_*υ *_come from modeling of the interfacial flux and are the film heat transfer coefficient and the interfacial area per unit volume, respectively. *k*_*ββ *_and *k*_*σσ *_are the effective thermal conductivities of the *β *and *σ-*phases, respectively; *k*_*βσ *_and *k*_*σβ *_are the coupling (cross) effective thermal conductivities between the two phases.

Rewriting Equations (8) and (9) in their operator form, we obtain

(10) $$\left[\begin{array}{cc} \gamma_{\beta }\frac{\partial }{\partial t}-k_{\beta \beta }\Delta +h & -k_{\beta \sigma }\Delta -ha_{\upsilon }\\ -k_{\sigma \beta }\Delta -ha_{\upsilon } & \gamma_{\sigma }\frac{\partial }{\partial t}-k_{\sigma \sigma }\Delta +ha_{\upsilon } \end{array}\right]\left[\begin{array}{c} T_{\beta }\\ T_{\sigma } \end{array}\right]=0$$

An uncoupled form can then be obtained by evaluating the operator determinant such that

(11) $$\left[\left(\gamma_{\beta }\frac{\partial }{\partial t}-k_{\beta \beta }\Delta +ha_{\upsilon }\right)\left(\gamma_{\sigma }\frac{\partial }{\partial t}-k_{\sigma \sigma }\Delta +ha_{\upsilon }\right)-{\left(k_{\beta \sigma }\Delta -ha_{\upsilon }\right)}^{2}\right]{\langle T_{i}\rangle }^{i}=0$$

where the index *i *can take *β *or *σ*. Its explicit form reads, after dividing by *ha*_υ_(*γ*_*β *_+ *γ*_*σ*_)

(12) $$\frac{\partial T_{i}}{\partial t}+\tau_{q}\frac{\partial^{2}T_{i}}{\partial t^{2}}=\alpha \Delta T_{i}+\alpha \tau_{T}\frac{\partial }{\partial t}\left(\Delta T_{i}\right)+\frac{\alpha }{k}\left[F(\text{r},t)+\tau_{q}\frac{\partial F(\text{r},t)}{\partial t}\right]$$

where

(13) $$\begin{array}{l} \tau_{q}=\frac{\gamma_{\beta }\gamma_{\sigma }}{ha_{\upsilon }(\gamma_{\beta }+\gamma_{\sigma })},\;\tau_{T}=\frac{\gamma_{\beta }k_{\sigma \sigma }+\gamma_{\sigma }k_{\beta \beta }}{ha_{\upsilon }(k_{\beta \beta }+k_{\sigma \sigma }+k_{\beta \sigma }+k_{\sigma \beta })},\\ k=k_{\beta \beta }+k_{\sigma \sigma }+k_{\beta \sigma }+k_{\sigma \beta },\;\alpha =\frac{k_{\beta \beta }+k_{\sigma \sigma }+k_{\beta \sigma }+k_{\sigma \beta }}{\gamma_{\beta }+\gamma_{\sigma }},\\ F(\text{r},t)+\tau_{q}\frac{\partial F(\text{r},t)}{\partial t}=\frac{k_{\beta \sigma }k_{\sigma \beta }-k_{\beta \beta }k_{\sigma \sigma }}{ha_{\upsilon }}\Delta^{2}T_{i}. \end{array}$$

Equation (12) is not a classical heat-conduction equation, but can be regarded as a dual-phase-lagging (DPL) heat-conduction equation with ((*k*_*βσ*_*k*_*σβ *_- *k*_*ββ*_*k*_*σσ*_)/(*ha*_*υ*_))Δ^2^*T*_*i *_as the DPL source-related term $F(\text{r},t)+\tau_{q}\frac{\partial F(\text{r},t)}{\partial t}$ and with *τ*_*q *_and *τ*_*T *_as the phase lags of the heat flux and the temperature gradient, respectively [2,18,69]. Here, *F*(**r**,*t*) is the volumetric heat source. *k*, *ρc*, and *α *are the effective thermal conductivity, capacity and diffusivity of nanofluids, respectively.

The computations of *k*_*ββ*_, *k*_*σσ*_, *k*_*βσ*_, and *k*_*σβ *_are available in [27,28] for some typical nanofluids. The coupled-transport contribution to the nanofluid thermal conductivity, the term (*k*_*βσ *_+ *k*_*σβ*_), can be as high as 10% of the of the overall thermal conductivity [27,28]. The more striking effect of the coupled transport on nanofluid heat conduction can be found by considering

(14) $$\frac{\tau_{T}}{\tau_{q}}=1+\frac{\gamma_{\beta }^{2}k_{\sigma \sigma }+\gamma_{\sigma }^{2}k_{\beta \beta }-2\gamma_{\beta }\gamma_{\sigma }k_{\beta \sigma }}{\gamma_{\beta }\gamma_{\sigma }(k_{\beta \beta }+k_{\sigma \sigma }+k_{\beta \sigma }+k_{\sigma \beta })},$$

which is smaller than 1 when

(15) $$\gamma_{\beta }^{2}k_{\sigma \sigma }+\gamma_{\sigma }^{2}k_{\beta \beta }-2\gamma_{\beta }\gamma_{\sigma }k_{\beta \sigma }={\left(\gamma_{\beta }\sqrt{k_{\sigma \sigma }}-\gamma_{\sigma }\sqrt{k_{\beta \beta }}\right)}^{2}+2\gamma_{\beta }\gamma_{\sigma }(\sqrt{k_{\beta \beta }k_{\sigma \sigma }}-k_{\beta \sigma })<0.$$

Therefore, by the condition for the existence of thermal waves that requires *τ*_*T*_/*τ*_*q*_*<*1 [18,70], thermal waves may be present in nanofluid heat conduction.

Note also that, for heat conduction in nanofluids, there is a time-dependent source term *F*(**r**,*t*) in the DPL heat conduction (Equations (12) and (13)). Therefore, the resonance can also occur. When *k*_*βσ *_= *k*_*σβ *_= 0 so that *τ*_*T*_/*τ*_*q *_is always larger than 1, thermal waves and resonance would not appear. Therefore, the coupled transport could change the nature of heat conduction in nanofluids from a diffusion process to a wave process, thus having a significant effect on nanofluid heat conduction.

Therefore, the cross coupling between the heat conduction in the fluid and particle manifests itself as thermal waves at the macroscale. Depending on factors such as material properties of nanoparticles and base fluids, nanoparticles' geometrical structure and their distribution in the base fluids, and interfacial properties and dynamic processes on particle-fluid interfaces, the cross-coupling-induced thermal waves may either enhance or counteract with the molecular-dynamics-driven heat diffusion. Consequently, the heat conduction may be enhanced or weakened by the presence of nanoparticles. This explains the thermal conductivity data that fall outside the H-S bounds (Figures 1 and 2).

If the coupled transport between heat conduction and particle diffusion is considered, then the temperature *T *and particle volume fraction *φ *satisfy the following equations of energy and mass conservation:

(16) $$\gamma_{\beta }\frac{\partial T_{\beta }}{\partial t}=k_{\beta \beta }\Delta T_{\beta }+k_{\beta \sigma }\Delta T_{\sigma }+\rho_{\sigma }k_{\beta \text{m}}\Delta \varphi +ha_{\upsilon }\left(T_{\sigma }-T_{\beta }\right),$$

(17) $$\gamma_{\sigma }\frac{\partial T_{\sigma }}{\partial t}=k_{\sigma \sigma }\Delta T_{\sigma }+k_{\sigma \beta }\Delta T_{\beta }+\rho_{\sigma }k_{\sigma \text{m}}\Delta \varphi -ha_{\upsilon }\left(T_{\sigma }-T_{\beta }\right),$$

and

(18) $$\rho_{\sigma }\frac{\partial \varphi }{\partial t}=\rho_{\sigma }D_{\sigma \sigma }\Delta \varphi +D_{\text{m}\beta }\Delta T_{\beta }+D_{m\sigma }\Delta T_{\sigma }+D_{\text{mT}}\left(T_{\beta }-T_{\sigma }\right),$$

where subscripts m and T stand for mass transport and thermal transport, respectively. *D*_*σσ *_is the effective diffusion coefficient for nanoparticles. *k*_*β*m_, *k*_*σ*m_, *D*_m*β*_, *D*_m*σ*_, and *D*_mT _are five transport coefficients for coupled heat and mass transport. By following a similar procedure as that of developing Equation (12), an uncoupled form with *u *(*T*_*β*_, *T*_*σ*_, or *φ*) as the sole unknown variable is obtained,

(19) $$\frac{\partial \left(\Delta u\right)}{\partial t}+\tau_{q}\frac{\partial^{2}\left(\Delta u\right)}{\partial t^{2}}=\alpha \Delta \left(\Delta u\right)+\alpha \tau_{T}\frac{\partial }{\partial t}\left(\Delta \left(\Delta u\right)\right)+\frac{\alpha }{k}\left[F(\text{r},t)+\tau_{q}\frac{\partial F(\text{r},t)}{\partial t}\right]$$

where

(20) $$\tau_{q}=\frac{\gamma_{\beta }k_{\sigma \sigma }+\gamma_{\sigma }k_{\beta \beta }+\gamma_{\beta }\gamma_{\sigma }D_{\sigma \sigma }}{D_{\text{mT}}\left(\gamma_{\sigma }k_{\beta \text{m}}-\gamma_{\beta }k_{\sigma \text{m}}\right)+ha_{\upsilon }\left(k_{\beta \beta }+k_{\beta \sigma }+k_{\sigma \beta }+k_{\sigma \sigma }\right)+ha_{\upsilon }D_{\sigma \sigma }\left(\gamma_{\beta }+\gamma_{\sigma }\right)},$$

(21) $$\begin{array}{l} k=\frac{\gamma_{\beta }+\gamma_{\sigma }}{D_{\text{mT}}\left(\gamma_{\sigma }k_{\beta \text{m}}-\gamma_{\beta }k_{\sigma \text{m}}\right)+ha_{\upsilon }\left(k_{\beta \beta }+k_{\beta \sigma }+k_{\sigma \beta }k_{\sigma \sigma }\right)+ha_{\upsilon }D_{\sigma \sigma }\left(\gamma_{\beta }+\gamma_{\sigma }\right)}\\ \times \left\{k_{\beta \text{m}}\left[D_{\text{mT}}\left(k_{\sigma \beta }+k_{\sigma \sigma }\right)-ha_{\upsilon }\left(D_{\text{m}\beta }+D_{\text{mT}}\right)\right]-k_{\sigma \text{m}}\left[D_{\text{mT}}\left(k_{\beta \beta }+k_{\beta \sigma }\right)+ha_{\upsilon }\left(D_{\text{m}\sigma }+D_{\text{m}\beta }\right)\right]\right\}\\ \times \frac{ha_{\upsilon }D_{\sigma \sigma }\left(\gamma_{\beta }+\gamma_{\sigma }\right)\left(k_{\beta \beta }+k_{\beta \sigma }+k_{\sigma \beta }+k_{\sigma \sigma }\right)}{D_{\text{mT}}\left(\gamma_{\sigma }k_{\beta \text{m}}-\gamma_{\beta }k_{\sigma \text{m}}\right)+ha_{\upsilon }\left(k_{\beta \beta }+k_{\beta \sigma }+k_{\sigma \beta }k_{\sigma \sigma }\right)+ha_{\upsilon }D_{\sigma \sigma }\left(\gamma_{\beta }+\gamma_{\sigma }\right)} \end{array}$$

(22) $$\alpha =\frac{k}{\gamma_{\beta }+\gamma_{\sigma }}$$

(23) $$\begin{array}{l} \frac{1}{\tau_{T}}=\frac{k_{\beta \text{m}}\left[D_{\text{mT}}\left(k_{\sigma \beta }+k_{\sigma \sigma }\right)-ha_{\upsilon }\left(D_{\text{m}\beta }+D_{\text{mT}}\right)\right]-k_{\sigma \text{m}}\left[D_{\text{mT}}\left(k_{\beta \beta }+k_{\beta \sigma }\right)+ha_{\upsilon }\left(D_{\text{m}\sigma }+D_{\text{m}\beta }\right)\right]}{D_{\sigma \sigma }\left(\gamma_{\beta }k_{\sigma \sigma }-\gamma_{\sigma }k_{\beta \beta }\right)+k_{\beta \beta }k_{\sigma \sigma }-k_{\beta \sigma }k_{\sigma \beta }-\gamma_{\sigma }k_{\beta \text{m}}D_{\text{m}\beta }-\gamma_{\beta }k_{\sigma \text{m}}D_{\text{m}\sigma }}\\ \times \frac{ha_{\upsilon }D_{\sigma \sigma }\left(k_{\beta \beta }+k_{\beta \sigma }+k_{\sigma \beta }+k_{\sigma \sigma }\right)}{D_{\sigma \sigma }\left(\gamma_{\beta }k_{\sigma \sigma }-\gamma_{\sigma }k_{\beta \beta }\right)+k_{\beta \beta }k_{\sigma \sigma }-k_{\beta \sigma }k_{\sigma \beta }-\gamma_{\sigma }k_{\beta \text{m}}D_{\text{m}\beta }-\gamma_{\beta }k_{\sigma \text{m}}D_{\text{m}\sigma }} \end{array}$$

(24) $$\begin{array}{l} F(\text{r},t)+\tau_{q}\frac{\partial F(\text{r},t)}{\partial t}=\\ \frac{ha_{\upsilon }{\left(\gamma_{\beta }+\gamma_{\sigma }\right)}^{2}}{D_{\text{mT}}\left(\gamma_{\sigma }k_{\beta \text{m}}-\gamma_{\beta }k_{\sigma \text{m}}\right)+ha_{\upsilon }\left(k_{\beta \beta }+k_{\beta \sigma }+k_{\sigma \beta }+k_{\sigma \sigma }\right)+ha_{\upsilon }D_{\sigma \sigma }\left(\gamma_{\beta }+\gamma_{\sigma }\right)}\frac{\partial^{2}u}{\partial t^{2}}\\ +\frac{\gamma_{\beta }\gamma_{\sigma }\left(\gamma_{\beta }+\gamma_{\sigma }\right)}{D_{\text{mT}}\left(\gamma_{\sigma }k_{\beta \text{m}}-\gamma_{\beta }k_{\sigma \text{m}}\right)+ha_{\upsilon }\left(k_{\beta \beta }+k_{\beta \sigma }+k_{\sigma \beta }+k_{\sigma \sigma }\right)+ha_{\upsilon }D_{\sigma \sigma }\left(\gamma_{\beta }+\gamma_{\sigma }\right)}\frac{\partial^{3}u}{\partial t^{3}}\\ +\frac{\left(\gamma_{\beta }+\gamma_{\sigma }\right)\left[k_{\beta \text{m}}\left(k_{\sigma \sigma }D_{\text{m}\beta }-k_{\sigma \beta }D_{\text{m}\sigma }\right)+k_{\sigma \text{m}}\left(k_{\beta \beta }D_{\text{m}\sigma }-k_{\beta \sigma }D_{\text{m}\beta }\right)\right]}{D_{\text{mT}}\left(\gamma_{\sigma }k_{\beta \text{m}}-\gamma_{\beta }k_{\sigma \text{m}}\right)+ha_{\upsilon }\left(k_{\beta \beta }+k_{\beta \sigma }+k_{\sigma \beta }+k_{\sigma \sigma }\right)+ha_{\upsilon }D_{\sigma \sigma }\left(\gamma_{\beta }+\gamma_{\sigma }\right)}\Delta^{3}u\\ -\frac{D_{\sigma \sigma }\left(\gamma_{\beta }+\gamma_{\sigma }\right)\left(k_{\beta \beta }k_{\sigma \sigma }-k_{\beta \sigma }k_{\sigma \beta }\right)}{D_{\text{mT}}\left(\gamma_{\sigma }k_{\beta \text{m}}-\gamma_{\beta }k_{\sigma \text{m}}\right)+ha_{\upsilon }\left(k_{\beta \beta }+k_{\beta \sigma }+k_{\sigma \beta }+k_{\sigma \sigma }\right)+ha_{\upsilon }D_{\sigma \sigma }\left(\gamma_{\beta }+\gamma_{\sigma }\right)}\Delta^{3}u \end{array}$$

This can be regarded as a DPL heat-conduction equation regarding Δ*u *with *τ*_*q*_*, τ*_*T*_, and $F(\text{r},t)+\tau_{q}\frac{\partial F(\text{r},t)}{\partial t}$ as the phase lags of the heat flux and the temperature gradient, and the source-related term, respectively. Therefore, the coupled heat and mass transport is capable of varying not only thermal conductivity from that in Equation (13) to the one in Equation (21) but also the nature of heat conduction from that in Equation (12) to the one in Equation (19). As practical nanofluid system always involves many transport processes simultaneously, the coupled transport could play a significant role. For assessing its effect and understanding heat conduction in nanofluids, future research is in great demand on coupling (cross) transport coefficients that are derivable by approaches like the up-scaling with closures [2,27,28], the kinetic theory [71,72], the time-correlation functions [73,74], and the experiments based on phenomenological flux relations [68]. While the uncoupled form of conservation equations, such as Equations (12) and (19), is very useful for examining nature of heat transport, its coupled form, such as Equations (8), (9), (16)-(18), is normally more readily to be resolved for the temperature or concentration fields after all the transport coefficients are available.

#### Brownian motion

In nanofluids, nanoparticles randomly move through liquid and possibly collide. Such a Brownian motion was thus proposed to be one of the possible origins for thermal conductivity enhancement because (i) it enables direct particle-particle transport of heat from one to another, and (ii) it induces surrounding fluid flow and thus so-called microconvection. The ratio of the former contribution to the thermal conductivity (*k*_BD_) to the base fluid conductivity (*k*_f_) is estimated based on the kinetic theory [75],

(25) $$\frac{k_{\text{BD}}}{k_{\text{f}}}=\frac{{\left(\rho c\right)}_{\text{p}}\varphi k_{\text{B}}T}{3\pi \mu d_{\text{p}}k_{\text{f}}}$$

where subscripts p and BD stand for the nanoparticle and the Brownian diffusion, respectively; *k*_B _is the Boltzmann's constant (1.38065 × 10^-23^J/K); and *μ *is the fluid viscosity. The kinetic theory also gives an upper limit for the ratio of the latter's contribution to the thermal conductivity (*k*_BC_) to the base fluid conductivity (*k*_f_) [76],

(26) $$\frac{k_{\text{BC}}}{k_{\text{f}}}=\frac{k_{\text{B}}T}{3\pi \mu d_{\text{p}}\alpha_{\text{f}}}$$

where subscript BC refers to the Brownian-motion-induced convection, and *α*_f _is the thermal diffusivity of the base fluid.

Consider a 1% volume fraction of *d*_p _= 10 nm copper nanoparticle in water suspension at *T *= 300 K. (*ρc*)_P _= 8900 kg/m^3 ^× 0.386 kJ/(kg K) = 3435.4 kJ/(m^3 ^K), μ = 0.798 × 10^-3^kg/(ms), *k*_f _= 0.615 W/(mK), and *α*_f _= 1.478 × 10^-7 ^m^2^/s. These yield *k*_BD_/*k*_f _= 3.076 × 10^-6 ^and *k*_BC_/*k*_f _= 3.726 × 10^-4^. Therefore, both contributions are negligibly small.

Although the direct contribution of particle Brownian motion to the nanofluid conductivity is negligible, its indirect effect could be significant because it plays an important role in processes of particle aggregating and coupled transport.

## Concluding remarks

Under the specified volume fractions and thermal conductivities of the two phases in the colloidal state, the interfacial energy transport between the two phases favors a configuration in which the higher-conductivity phase forms a continuous path for thermal flow and disperses the lower-conductivity phase. The effective thermal conductivity is thus bounded by those corresponding to the two limiting morphologies: the well-dispersed configuration of the higher-conductivity phase in the lower-conductivity phase and the well-dispersed configuration of the lower-conductivity phase in the higher-conductivity phase, corresponding to the lower and the upper bounds of thermal conductivity, respectively. Without considering the effect of interfacial resistance and cross coupling among various transport processes, the classical effective-medium theory gives these bounds known as the H-S bounds. A wide separation of these two bounds offers spacious room of manipulating nanofluid thermal conductivity via the morphology effect.

In a nanofluid system, there are normally two or more transport processes that occur simultaneously. The cross coupling among these processes causes new induced effects of flows occurring without or against its primary thermodynamic driving force and is capable of changing the nature of heat conduction via inducing thermal waves and resonance. Depending on the microscale physics (factors like material properties of nanoparticles and base fluids, nanoparticles' morphology in the base fluids, and interfacial properties and dynamic processes on particle-fluid interfaces), the heat diffusion and thermal waves may either enhance or counteract each other. Consequently, the heat conduction may be enhanced or weakened by the presence of nanoparticles.

The direct contributions of ordered liquid layer and particle Brownian motion to the nanofluid conductivity are negligible. Their influence on the particle morphology and/or the coupled transport could, however, offer a strong indirect effect to the nanofluid conductivity.

Therefore, nanofluids with conductivity of upper H-S bound can be obtained by manipulating particles into an interconnected configuration that disperses the base fluid, and thus significantly enhancing the particle-fluid interfacial energy transport. Nanofluids with conductivity higher than the upper H-S bound could also be developed by manipulating the cross coupling among various transport processes and thus the nature of heat conduction in nanofluids.

## Abbreviations

DPL: dual-phase-lagging; H-S: Hashin-Shtrikman.

## Competing interests

The authors declare that they have no competing interests.

## Authors' contributions

Both authors contributed equally.
